# Accumulation of advanced glycation end products evaluated by skin autofluorescence and incident frailty in older adults from the Bordeaux Three-City cohort

**DOI:** 10.1371/journal.pone.0186087

**Published:** 2017-10-17

**Authors:** Sophie Pilleron, Kalina Rajaobelina, Maturin Tabue Teguo, Jean-François Dartigues, Catherine Helmer, Cécile Delcourt, Vincent Rigalleau, Catherine Féart

**Affiliations:** 1 Univ. Bordeaux, Inserm, Bordeaux Population Health Research Center, team LEHA, UMR 1219, Bordeaux, France; 2 CHU de Bordeaux, Service de Nutrition-Diabétologie, Hôpital Haut-Lévêque, Pessac, France; Institute of Nutrition, GERMANY

## Abstract

**Aim:**

We analyzed the cross-sectional and prospective relationships between the accumulation of advanced glycation end products (AGE), assessed by skin autofluorescence (AF) and frailty and its components.

**Methods:**

A total of 423 participants of the Bordeaux sample of the Three-City study 75 years of age or older in 2009–2010 were included in the cross-sectional analysis. Among them, 255 initially non-frail participants were re-examined 4 years later. Skin AF (arbitrary units (AU)) was measured using the AGE Reader. Frailty was defined using Fried’s criteria. Associations were assessed with logistic regression models.

**Results:**

Mean skin AF at baseline was 2.81 ±0.68 AU and 16.8% participants were frail. Adjusted for sociodemographic and health characteristics, skin AF was associated neither with prevalent frailty as a whole (Odds Ratio (OR) = 1.2; 95% Confidence Interval: 0.8–1.9) nor with any of its components. Among 255 non-frail participants, 32 became frail over 4 years. In multivariate analyses, skin AF was not associated with incident frailty as a whole (OR = 1.0; 0.5–2.0) but with a doubled risk of incident exhaustion (OR = 2.0; 1.2–3.6) and low energy expenditure (OR = 2.0; 1.1–3.7). No association was observed with other criteria.

**Conclusion:**

In French older community-dwellers aged 75 years and over, the accumulation of AGEs evaluated by skin AF was not associated with prevalent or incident frailty but with the 4-year risk of exhaustion and low energy expenditure. Further studies with larger samples are needed to confirm our results.

## Introduction

Advanced glycation end products (AGEs) are produced by the glycation of proteins, lipids, and nucleic acids that can cause widespread tissue and cellular damages [1]. AGEs accumulate during normal aging [1] but in an excessive way in diabetes [2] and in chronic kidney disease [3]. AGEs are involved in the phenomenon of metabolic memory that refers to the long-term effect of previous poor metabolic environment [4]. Their accumulation could have an important role in frailty [5] and their accumulation in the skeletal muscle tissue is suggested as one of the causes of the reduction of muscle and physical function with aging [6].

Frailty is a reversible state associated with higher risk of dependence, institutionalization, morbidity and mortality [7]. Prevalence of frailty in older community-dwelling adults varies dramatically from 4 to 59% according to the definition used [8].

A few studies investigated the relationship between circulating AGEs and frailty in older community-dwelling adults [9–11], but circulating AGEs fluctuate physiologically. Measurement of tissue-bound AGEs seems more suitable for AGEs assessment, notably in tissues with a slow turnover such as dermis [12]. Skin autofluorescence (skin AF) is a non-invasive marker of the accumulation of AGEs in long-lived proteins [13]. To date, a single cross-sectional study has shown that skin AF was independently associated with grip strength and leg extension power in adult men [14].

No studies have investigated the association between skin AF and frailty or its components in older adults yet. We therefore examined the cross-sectional and prospective relationship of the accumulation of AGEs assessed by skin AF with frailty in French older community-dwellers 75 years of age or older.

## Materials and methods

### Study population

We used data of the Bordeaux sample of the Three-City (3C) study, a multicenter population-based cohort of community-dwellers 65 years of age or older started in 1999–2000 that aimed at estimating the risk of dementia attributable to vascular factors. Details are described elsewhere [15]. All participants gave their written informed consent. Data were collected using standardized questionnaires at baseline and follow-ups (wave 1 in 2001–2002, wave 2 in 2003–2004, wave 3 in 2006–2007, wave 4 in 2009–2010, wave 5 in 2011–2012 and wave 6 in 2013–2014). The 3C study has been approved by the ethical committees of the Kremlin-Bicêtre University Hospital (Paris) and Sud-Mediterranée II. The present analysis is based on data of waves 4 and 6 since definition of frailty was strictly comparable.

Of 1,214 participants seen in wave 4 (hereinafter referred to as baseline), 457 had complementary examinations including skin AF measurement. Among them, 433 had data on frailty status at baseline. We excluded 10 participants with missing data for covariates, remaining 423 for the cross-sectional analysis.

Of these, we excluded 71 prevalent frail participants, 42 deceased, 41 who were not seen at wave 6 and 14 with insufficient data to identify frailty in wave 6, leaving 255 for the prospective analysis.

### Skin autofluorescence (skin AF)

At baseline, skin AF expressed as arbitrary units (AU) has been measured in triplicate at the skin site on the inner forearm–body part easily accessible and little exposed to the sun—using the AGE Reader (DiagnOptics Technologies B.V., Groningen, Netherlands) at Bordeaux University hospital [13]. The average of the three values was used in the analysis. Skin AF measurement was validated against skin biopsies and values were correlated with glycated collagen, pentosidine and Nε-carboxymethyl-lysine (CML) levels in healthy individuals, but also in Type 2 and Type 1 diabetes patients [13].

### Frailty

According to Fried’s definition, participants were frail if they met 3 or more criteria among the following 5 self-reported criteria [7]: (i) shrinking; (ii) exhaustion; (iii) weakness; (iv) slowness; (v) low energy expenditure.

We defined shrinking (i) as the self-reported recent unintentional loss of 3 kg or more. In case of missing data (n = 5), this criteria was considered as fulfilled if body mass index (BMI) was <21 kg/m^2^. Exhaustion (ii) was defined using two items of the Center for Epidemiologic Studies Depression Scale (CES-D) [16]. Respondents were considered as exhausted if they answered yes to at least one of the two following items: During the past week, “I felt that everything I did was an effort” and “I could not get going”. Weakness (iii) was defined using the weakest quintile stratified by body mass index (BMI) and sex of the handgrip strength. Slowness (iv) was ascertained using the slowest quintile stratified by height and sex of the 4-meter gait speed. Respondents unable to complete the respective physical performance tests were included as weak and as slow. Low energy expenditure (v) was defined as reporting no engagement in physical activities (strenuous leisure activities or sport).

### Other data

Baseline sociodemographic information included age, sex and education level (in two classes: low education level defined as no schooling or no diploma *versus* higher level). Smoking status was evaluated in 1999–2001 and categorized in “non-smokers” and “ex- or current smokers”. Polymedication was defined as participants taking ≥5 drugs at least once a week during the last month. Global cognitive performance was assessed using the Mini-Mental State Examination (MMSE) [17]. Depressive symptomatology was defined as a CES-D score ≥23 for women and ≥17 for men [16,18]. Diabetes mellitus (mainly Type 2) was defined as current use of antidiabetic drugs and/or fasting plasma glucose ≥7 mmol/L [19]. Estimated glomerular filtration rate (eGFR) was estimated using the modification of diet in renal disease study equation [20]. Chronic kidney disease (CKD) was defined as eGFR < 60 mL/ min/1.73 m^2^ [20].

### Data analysis

Univariate associations were tested using Student’t test or ANOVA for normally distributed quantitative variables (skin AF), Wilcoxon test for non-normally distributed variables (age and MMSE) and χ^2^ test or Fisher exact test for categorical variables.

The multivariate associations between skin AF, prevalent frailty and the 4-year risk of frailty were investigated using a logistic regression model adjusted for age, sex, education level, smoking status, polymedication, depressive symptomatology, MMSE, diabetes mellitus and CKD.

Separate multivariate models were run with each frailty component as outcome in participants who did not fulfill the analyzed frailty component at baseline.

In all models, skin AF, age and MMSE were kept as continuous variables since log-linearity assumption could not be rejected.

Statistical analyses were performed with SAS Statistical package release 9.3 (SAS institute Inc., Cary, NC, USA).

## Results

### Cross-sectional association

The sample consisted of 423 participants (63.4% females; median age = 81.9±6.0). Frailty status was statistically associated with higher age, lower MMSE, more depressive symptoms, and the presence of CKD (Table 1). Skin AF was higher in men, in ex- or current smokers, in polymedicated participants, in participants with diabetes mellitus, and in those with CKD (Table 2). Median skin AF did not differ statistically based on frailty status or its components (Table 3).

**Table 1:** Baseline characteristics of older adults based on prevalent and incident frailty, the Three-City study, Bordeaux, 2009–2010.

	Cross-sectional analysis					Prospective analysis		
	Prevalent frailty					4-year incident frailty		
	Total	No	Yes	p	Total	No	Yes	p
**Sample**	423	352 (83.2)	71 (16.8)		255	223 (87.5)	32 (12.6)	
**Sociodemography**								
Age (years), median ±IQR	81.3±6.0	80.8 ±5.5	84.3 ±7.2	< .001	80.4 ±5.1	80.3 ±5.1	82.5 ±7.5	.002
Women, n (%)	268 (63.4)	217 (61.7)	51 (71.8)	.10	97 (38.0)	88 (39.5)	9 (28.1)	.22
Education level, n (%)	111 (26.2)	91 (25.9)	20 (28.2)	.69	69 (27.1)	56 (25.1)	13 (40.6)	.06
**Health**								
Ex- or current smoker	147 (34.8)	119 (33.8)	28 (38.4)	.36	84 (32.9)	77 (34.5)	7 (21.9)	.15
MMSE, median ±IQR	28.0 ± 2.0	28.0 ± 2.0	27.0 ± 3.0	.003	28.0 ± 2.0	28.0 ±2.0	28.0 ±2.5	.27
Depressive symptomatology, n (%)	43 (10.2)	19 (5.4)	24 (33.8)	< .001	16 (6.3)	11 (4.9)	5 (15.6)	.04
Polymedication^a^, n (%)	273 (64.5)	216 (61.4)	57 (80.3)	.002	153 (60.0)	129 (57.9)	24 (75.0)	.06
Diabetes mellitus, n (%)	64 (15.1)	48 (13.6)	16 (22.5)	.06	36 (14.1)	32 (14.4)	4 (12.5)	1.00
CKD, n (%)				.02^b^			13 (40.6)	.29 ^b^
No	209 (49.4)	184 (52.3)	25 (35.2)		129 (57.9)	15 (46.9)		
Yes	146 (34.5)	115 (32.7)	31 (43.7)		86 (33.7)	73 (32.7)		
Missing values	68 (16.1)	53 (15.1)	15 (21.1)		21 (9.4)	4 (12.5)		

Notes. Abbreviations: CKD: Chronic Kidney Disease; IQR: Interquartile range; MMSE: Mini-mental state examination; SD: standard deviation.

^a^ Polymedication ≥ 5 medications taken regularly

^b^ Missing values were not considered for χ^2^ test.

**Table 2:** Skin autofluorescence based on baseline characteristics of older adults in cross-sectional and prospective analysis samples, the Three-City study, Bordeaux, 2009–2010.

	Cross-sectional analysis sample			Prospective analysis sample		
	N	Mean ±SD	p	N	Mean ±SD	p
**Total sample**	423	2.81 ± 0.68		255	2.75 ± 0.62	
**Sociodemography**						
Age (years)			1.00			0.65
[75–80]	162	2.80 ± 0.63		113	2.78 ± 0.61	
[80–85]	161	2.81 ± 0.64		97	2.71 ± 0.63	
[85-max]	100	2.81 ± 0.73		45	2.73 ± 0.65	
Sex			0.02			0.07
Men	155	2.91 ± 0.72		97	2.84 ± 0.70	
Women	268	2.75 ± 0.61		158	2.69 ± 0.57	
Education			0.19			0.06
No diploma	111	2.88 ± 0.64		69	2.87 ± 0.60	
Elementary with diploma	312	2.78 ± 0.66		186	2.70 ± 0.63	
Health						
Smoking status			0.04			0.08
No smoker	276	2.76 ± 0.62		171	2.74 ± 0.57	
Ex or current smoker	147	2.90 ± 0.71		84	2.77 ± 0.72	
MMSE^a^		-0.03	0.54		-0.03	0.61
Depressive symptomatology			0.16			0.47
No	380	2.79 ± 0.65		239	2.75 ± 0.62	
Yes	43	2.94 ± 0.73		16	2.64 ± 0.64	
Polymedication ^b^			0.003			0.006
No	150	2.68 ± 0.65		102	2.62 ± 0.59	
Yes	273	2.88 ± 0.65		153	2.83 ± 0.63	
Diabetes mellitus			0.009			0.01
No	359	2.77 ± 0.64		219	2.70 ± 0.58	
Yes	64	3.00 ± 0.71		36	3.05 ± 0.77	
CKD			0.002			0.13
No	209	2.68 ± 0.66		144	2.69 ± 0.63	
Yes	146	2.91 ± 0.65		86	2.82 ± 0.61	

Notes. Abbreviations: CKD: Chronic Kidney Disease; MMSE: Mini-mental state examination; SD: standard deviation.

^a^Pearson coefficient.

^b^Polymedication ≥ 5 medications taken regularly.

**Table 3:** Skin autofluorescence based on prevalent and incident frailty and its components, the Three-City study, Bordeaux, 2009–2010.

	Prevalence cases					Incident cases				
	No		Yes			No		Yes		
	n	mean ±SD	n	mean ±SD	p	n^a^	mean ±SD	n^a^	mean ±SD	p
Frailty	352	2.79 ±0.64	71	2.92 ±0.73	0.11	223	2.75 ± 0.64	32	2.75 ± 0.49	0.96
Shrinking	397	2.80 ±0.65	37	2.90 ±0.67	0.38	289	2.74 ± 0.63	7	2.66 ± 0.42	0.72
Exhaustion	354	2.79 ±0.65	70	2.92 ±0.68	0.13	183	2.68 ± 0.63	42	3.03 ± 0.56	0.001
Weakness	292	2.77 ±0.63	96	2.82 ±0.68	0.50	183	2.72 ± 0.60	29	2.81 ± 0.50	0.42
Slowness	326	2.78 ±0.65	108	2.90 ±0.67	0.10	188	2.72 ± 0.61	60	2.71 ± 0.66	0.86
Low energy expenditure	188	2.75 ±0.67	241	2.86 ±0.64	0.09	74	2.60 ± 0.62	76	2.84 ± 0.64	0.020

^a^ sample size including only non-prevalent frailty cases at baseline.

Multivariate analyses showed no statistically significant association with frailty or its components in model adjusted for sociodemographic, health covariates including diabetes mellitus and CKD (Table 4).

**Table 4:** Multivariate associations* between skin AF and prevalent and incident frailty and its components, the Three-City Study, Bordeaux.

	Cross-sectional analysis			Prospective analysis		
	n/N	OR (95%CI)	p	n/N	OR (95%CI)	p
Frailty	71/423	1.14 (0.73–1.77)	0.56	32/255	0.95 (0.45–1.98)	0.88
Unintentional weight loss	37/434	0.99 (0.57–1.71)	0.96	7/296	0.59 (0.14–2.51)	0.48
Exhaustion^$^	70/424	1.32 (0.88–1.99)	0.18	42/225	2.03 (1.16–3.58)	0.01
Weakness	96/388	1.15 (0.78–1.69)	0.48	30/212	1.24 (0.57–2.66)	0.59
Slowness	108/434	1.09 (0.75–1.58)	0.64	60/248	0.87 (0.51–1.48)	0.60
Low energy expenditure	241/429	1.16 (0.82–1.63)	0.41	76/150	1.99 (1.07–3.69)	0.03

Notes. n = number of incident cases; N = total sample.

* model adjusted for age, sex, education level, smoking status, polymedication, mini mental state examination, depressive symptomatology, diabetes mellitus and chronic kidney disease

^$^ To avoid collinearity issues (exhaustion is defined using 2 items of the Center for Epidemiologic Studies Depression Scale), model was not adjusted for depressive symptomatology defined by the Center for Epidemiologic Studies Depression Scale.

### Prospective association

The sample consisted of 255 non-frail participants. Median age was 80.4 ±5.1 and 62% were females.

Over the 4-year follow-up, 32 participants (12.5%) became frail. Incident frailty was statistically associated with higher age and the presence of depressive symptomatology (Table 1).

In the total sample, mean skin AF was 2.75 ± 0.62 AU and no correlation with age was found (Table 2). Statistically higher mean skin AF values were observed in polymedicated participants (p = 0.006) and in participants with diabetes mellitus (p = 0.01).

Mean skin AF did not differ statistically based on frailty status but was statistically higher in participants who reported exhaustion (p = 0.001) and low energy expenditure (p = 0.022) 4 years later (Table 3).

The multivariate analysis revealed no statistically significant association between skin AF and the 4-year risk of frailty (OR = 1.0; 95% Confidence Interval (CI): 0.5–1.9 –Table 4)). However, each 1-unit increase of skin AF doubled the risk of exhaustion (OR = 2.2; 95%CI: 1.2–3.7) and of low energy expenditure (OR = 1.9; 95%CI: 1.1–3.4).

## Discussion

For the first time, this study investigated the association between a non-invasive measurement of AGEs accumulation and the risk of frailty and its components in French older community-dwellers. Our findings revealed no association between skin AF and prevalent or incident frailty but with incident exhaustion and incident low energy expenditure.

Previous studies in older community-dwellers were based on circulating AGEs. In 559 moderately to severely disabled women 65 years of age or older from the Women’s Health and Aging Study I, elevated serum Nε-carboxymethyl-lysine (CML) was associated with poor grip strength [9]. In 944 participants 65 years of age or older from the InCHIANTI cohort, participants in the highest quartile of plasma CML had greater odds of slow walking speed [21]. In the Cardiovascular Health Study, odds of frailty increased with higher serum CML concentrations in men but the strength of the association was attenuated by adjustment for cognitive status, kidney function, and arthritis [11]. Direct comparisons with our findings are difficult notably because AGEs measurements’ methods are different.

The lack of association between skin AF and prevalent or incident frailty was surprising at first sight. However, several hypotheses could explain this negative finding. First, low sample size limited statistical power. Secondly, skin AF was described by a linear increase with age but this observation was restricted to subjects under the age of 70 [22]. Beyond this age, skin AF threshold may somehow be reached (*i*.*e*. illustrating a plateau effect) making our very old sample (81 years old on average) homogenous regarding skin AF. Moreover, we found no correlation between age and skin AF in both samples (r = 0.01; p = 0.77 in cross-sectional analysis sample and r = -0.02; p = 0.74 in prospective analysis sample). Lastly, a survival effect may be a third explanation. Indeed, people who reach the age 75 are somewhat survivors and frail individuals or those with higher skin AF may have died before reaching the age of 75.

Elevated skin AF values were associated with an increased 4 year-risk of exhaustion but not with prevalent exhaustion. This is congruent with existing literature. In the Fried’s conceptualization of frailty, subclinical diseases may play an etiologic role in frailty [23]. Exhaustion was thought to precede myocardial infarction and predicts severity of coronary artery diseases [24,25]. Skin AF is associated with atherosclerosis [26], elevated in stable coronary arteries diseases [27,28] and correlated with carotid intima media thickness [29]. Our findings also revealed an association with incident low energy expenditure and once again not with prevalent low energy expenditure. No observational studies have reported results about AGEs and physical activity so far. However, physical activity induced a statistically significant reduction of serum AGEs in interventional studies [30,31].

The lack of cross-sectional association but the existence of prospective association with exhaustion and low energy expenditure suggest that accumulation of AGEs is not a marker but a predictor of these two criteria whatever the initial frailty status.

Surprisingly, we found no association between skin AF and low grip strength or low gait speed while some arguments in literature were in favor of such an association. Elevated AGEs was associated with severe walking disability [32], and slow gait speed [21] and AGEs accumulate in skeletal muscle with aging in rats [33]. Skin AF was associated with low skeletal muscle index among middle-aged and older Japanese [34]. No evident hypothesis can explain the lack of association in our study. This warrants further investigations.

Our study has several limitations to keep in mind when interpreting our findings. First, fluorescence of non-AGEs tissue components could be detected, whereas non-fluorescent AGEs were not measured. However, validation studies have shown a good correlation between skin AF level and fluorescent and non-fluorescent AGEs from skin biopsies [13]. Secondly, our sample size is small leading to a limited statistical power, which could prevent detecting some additional significant associations. Low sample size precludes testing interaction with sex while some studies suggested more deleterious effect of AGE in women [35] and some other showed that higher circulating CML level was associated with frailty only in older men [11]. Lastly, each time we are interested in elderly people, we have to face to a probable selection bias due to survival effect. It was shown that older adults with elevated serum AGEs were at an increased risk of mortality [10]. This could lead to underestimation of observed association. However, the prevalence of frailty in our cross-sectional sample is close to frailty prevalence (17.8% (95% CI: 14.8–20.8)) observed in a representative sample of individuals aged 75 years old or older from the Bordeaux Three-City [36].

The strengths of our study are the use of cross-sectional and prospective and population-based design, the use of a validated non-invasive measurement of AGEs [13] and adjustment for major confounders, notably diabetes mellitus, CKD and cognitive performances.

In conclusion, this population-based study in community-dwellers aged 75 years or over suggested that the accumulation of AGEs is neither marker nor predictor of the occurrence of frailty as a whole 4 years later, but it is predictor of some of its component. These findings need to be confirmed by further studies with larger sample size and younger participants.
